# Left-Sided Abdominal Pain Revealing Acute Cholecystitis in Situs Inversus Totalis: Diagnostic Pitfalls and Surgical Considerations

**DOI:** 10.7759/cureus.103376

**Published:** 2026-02-10

**Authors:** José Emiliano González Flores, Aarón Gonzalez Espinosa

**Affiliations:** 1 Surgery, Tecnológico de Monterrey Campus Ciudad de Mexico, Mexico City, MEX; 2 General Surgery, Hospital General de México "Dr. Eduardo Liceaga", Mexico City, MEX

**Keywords:** abdominal pain, acute cholecystitis, diagnostic pitfalls, laparoscopic cholecystectomy, situs inversus totalis

## Abstract

Situs inversus totalis is a rare congenital condition that can complicate the diagnosis and management of acute abdominal pathology due to atypical symptom localization. Acute cholecystitis in this setting may present with left-sided abdominal pain, increasing the risk of misdiagnosis and delayed treatment. The scientific rationale for this case report is to emphasize the importance of recognizing anatomical variants when evaluating atypical abdominal presentations. We report the case of a 65-year-old woman who presented with left upper quadrant pain, nausea, and vomiting. Imaging studies demonstrated dextrocardia and complete mirror-image transposition of the thoracic and abdominal organs, consistent with situs inversus totalis, along with findings of acute calculous cholecystitis. The patient underwent laparoscopic cholecystectomy using a mirrored port placement technique without complications and had an uneventful recovery. This case highlights that early use of imaging and appropriate surgical adaptation enable safe laparoscopic management. Future research should focus on multicenter data to better characterize diagnostic delays and refine surgical strategies in patients with situs inversus totalis.

## Introduction

Situs inversus totalis is a rare congenital condition characterized by complete mirror-image transposition of the thoracic and abdominal organs, with an estimated prevalence of approximately one in 10,000 individuals [1,2]. Although most affected patients remain asymptomatic, this atypical anatomical configuration may significantly complicate the diagnosis and management of acute abdominal conditions. Acute cholecystitis, one of the most common causes of surgical abdominal pain, typically presents with right upper quadrant tenderness; however, in patients with situs inversus totalis, symptoms may manifest in the left upper quadrant, increasing the risk of misdiagnosis or delayed surgical management [3].

Early recognition of this anatomical variant is essential, as delayed diagnosis may lead to disease progression and potentially serious complications. Imaging modalities, including ultrasound and computed tomography, play a central role in establishing the diagnosis and guiding management. Laparoscopic cholecystectomy remains the treatment of choice, although safe execution requires technical adaptation to the mirrored anatomy and adherence to established principles for acute cholecystitis management [2,4].

We report the case of a 65-year-old woman with situs inversus totalis who presented with left-sided abdominal pain due to acute cholecystitis and was successfully treated with laparoscopic cholecystectomy.

The aim of this case report is to highlight key diagnostic pitfalls associated with acute cholecystitis in patients with situs inversus totalis and to discuss practical surgical considerations that enable safe laparoscopic management in this uncommon anatomical context.

## Case presentation

A 65-year-old woman with no relevant past medical history presented to the emergency department with a two-day history of progressive abdominal pain. The pain was predominantly localized to the left upper quadrant, colicky in nature, and associated with nausea and multiple episodes of postprandial vomiting. She denied fever, jaundice, or prior similar episodes.

On initial evaluation, the patient was tachycardic but hemodynamically stable. Physical examination revealed left upper quadrant tenderness with localized guarding. Laboratory studies showed leukocytosis with neutrophilia and mild transaminase elevation, with a slightly increased total bilirubin level.

**Figure 1:**
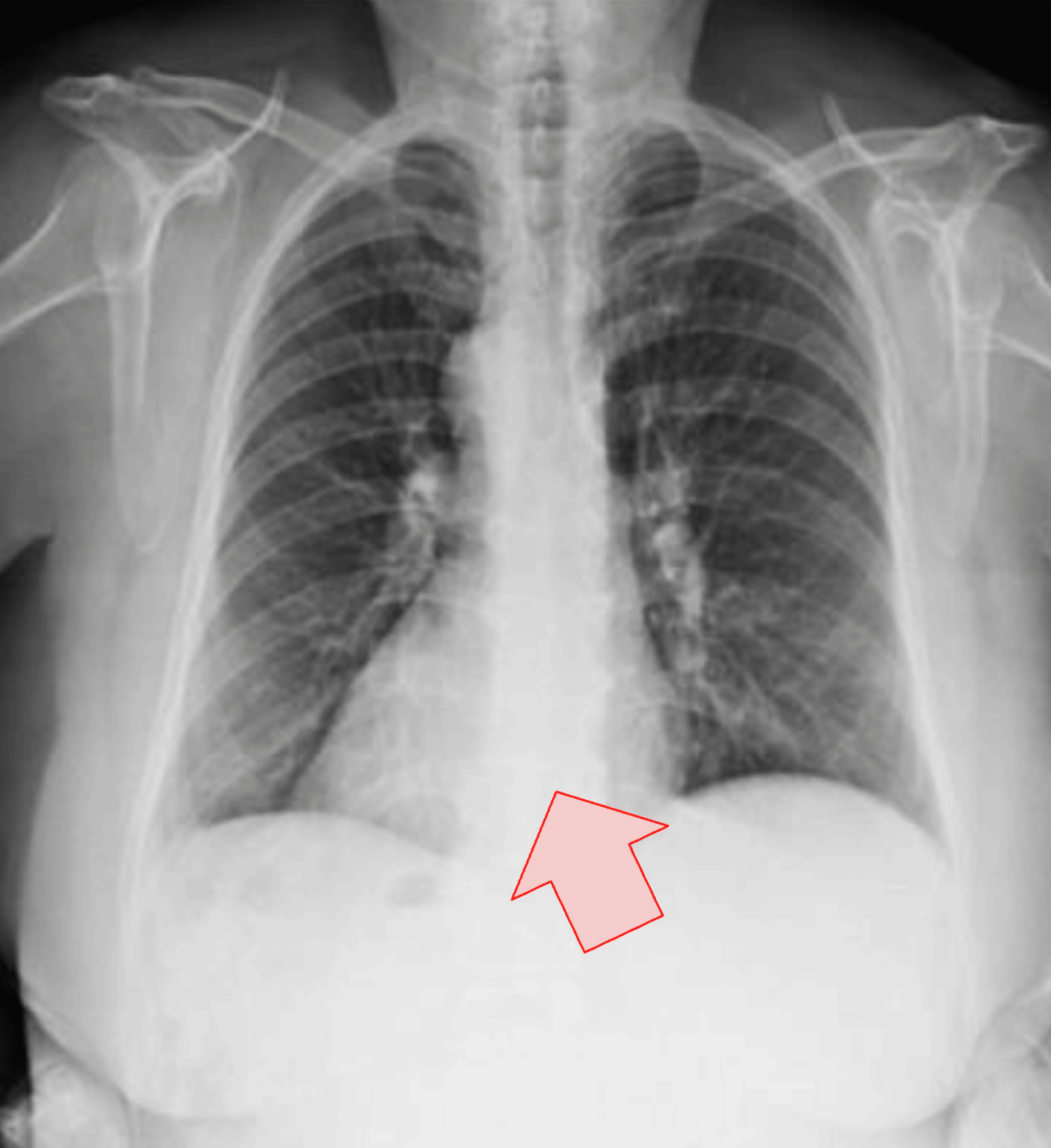
Chest radiograph demonstrating dextrocardia Posteroanterior chest radiograph showing the cardiac apex positioned on the right hemithorax (arrow), consistent with dextrocardia and suggestive of situs inversus totalis in the appropriate clinical context.

**Figure 2:**
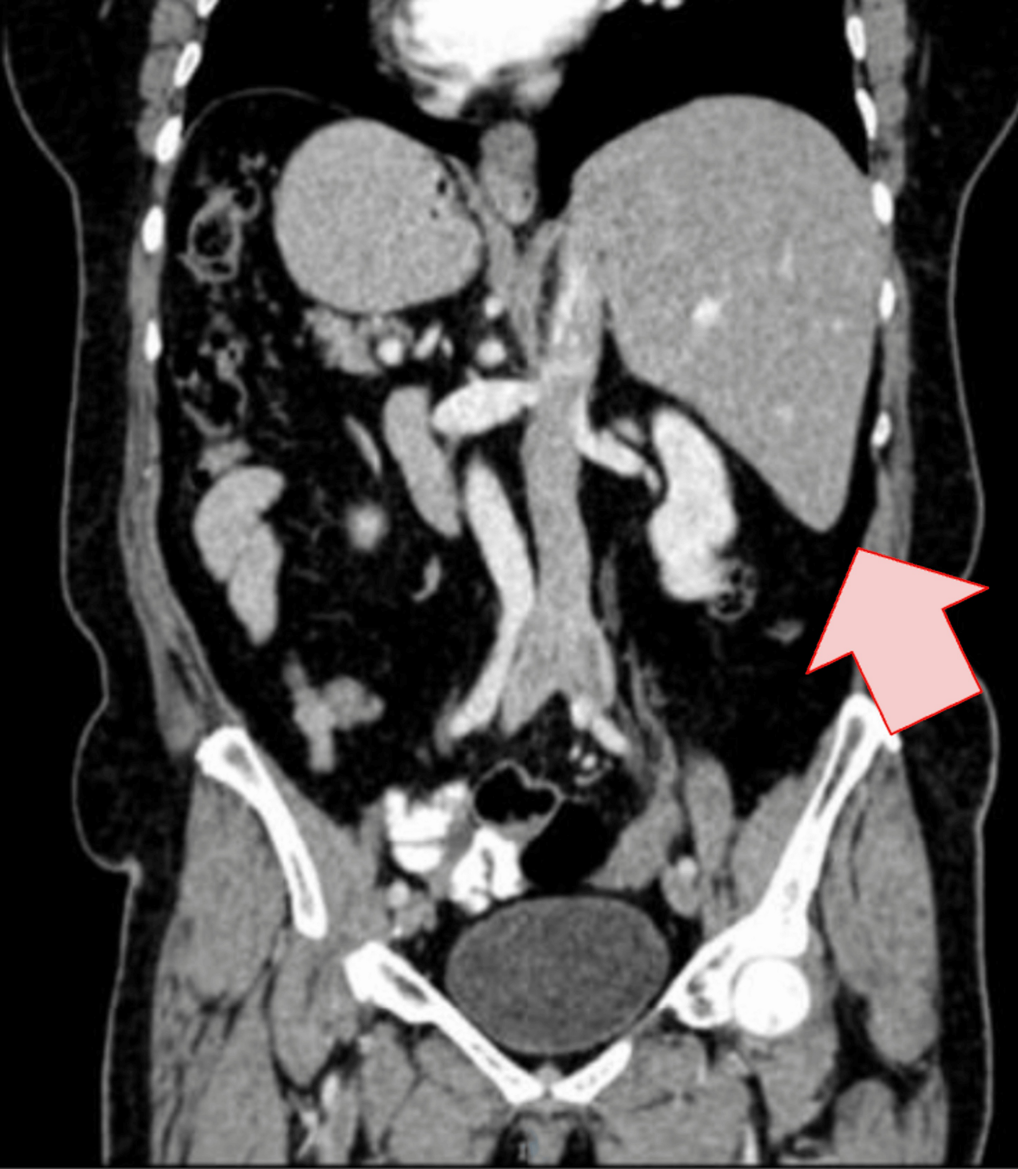
Contrast-enhanced abdominopelvic computed tomography demonstrating situs inversus totalis Coronal contrast-enhanced abdominopelvic computed tomography demonstrating mirror-image transposition of the abdominal viscera, with the liver located in the left upper quadrant (arrow), consistent with situs inversus totalis.

Chest radiography demonstrated dextrocardia, prompting suspicion of an underlying anatomical variant (Figure 1). Abdominal ultrasound identified a left-sided gallbladder with wall thickening, pericholecystic fluid, and a gallstone with posterior acoustic shadowing, findings consistent with acute cholecystitis. Contrast-enhanced computed tomography of the abdomen confirmed situs inversus totalis with complete mirror-image transposition of the thoracic and abdominal organs, as well as imaging features of acute calculous cholecystitis (Figure 2).

**Figure 3:**
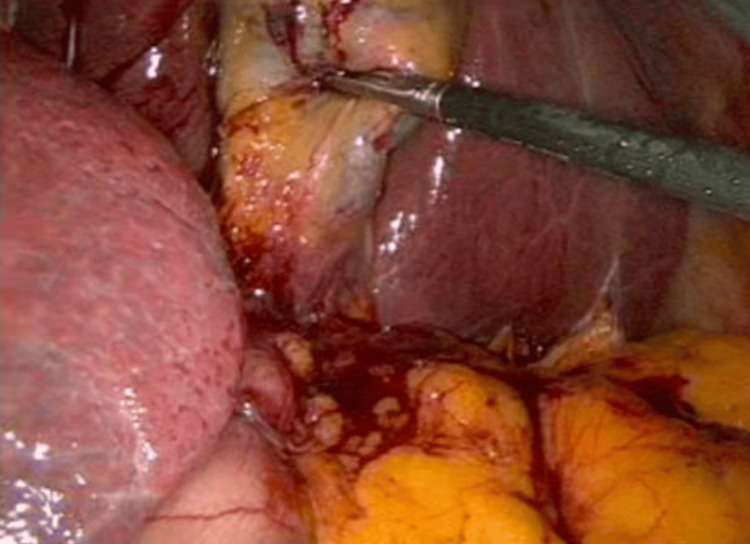
Laparoscopic view of the left-sided gallbladder in situs inversus totalis Intraoperative laparoscopic image demonstrating a left-sided gallbladder with exposure of Calot's triangle during laparoscopic cholecystectomy in a patient with situs inversus totalis.

Following imaging confirmation, the patient was initially managed with supportive care, including intravenous fluids and analgesia, and subsequently underwent laparoscopic cholecystectomy during the same admission using a mirrored port placement technique. Intraoperative findings included an inflamed and edematous gallbladder containing multiple calculi, with anatomy consistent with situs inversus totalis (Figure 3). The procedure was completed laparoscopically without intraoperative complications.

Postoperative recovery was uneventful, and the patient was discharged home within 48 hours in stable condition. At outpatient follow-up, she remained asymptomatic with no postoperative complications.

## Discussion

Situs inversus totalis represents a diagnostic challenge when acute abdominal pathology develops, as the mirror-image transposition of thoracic and abdominal organs may lead to atypical symptom localization and diagnostic delay. Acute cholecystitis classically presents with right upper quadrant pain; however, in patients with situs inversus totalis, left-sided abdominal pain may mislead clinicians toward alternative diagnoses, particularly in emergency settings [1,5]. This highlights the importance of maintaining a high index of suspicion when evaluating abdominal pain that does not follow typical anatomical patterns.

Imaging plays a pivotal role in establishing the diagnosis and preventing mismanagement. In the present case, chest radiography revealing dextrocardia prompted further investigation, while abdominal ultrasound and computed tomography confirmed both acute cholecystitis and situs inversus totalis. Cross-sectional imaging not only allows accurate diagnosis but also provides essential anatomical orientation for surgical planning, reducing the risk of intraoperative complications [6,7].

Laparoscopic cholecystectomy remains the treatment of choice for acute calculous cholecystitis in patients with situs inversus totalis and is not contraindicated by the anatomical variation [8]. Nevertheless, the mirrored anatomy requires technical adaptation, including reversed port placement and heightened awareness of altered spatial relationships within Calot's triangle. Strict adherence to established safety principles, particularly careful dissection and identification of critical structures, is essential to minimize the risk of bile duct injury [9,10].

This case underscores the importance of early recognition of situs inversus totalis, appropriate use of imaging, and thoughtful surgical adaptation. Awareness of this rare anatomical variant can prevent diagnostic delays and facilitate safe laparoscopic management, ultimately leading to favorable clinical outcomes. The key diagnostic and surgical lessons derived from this case are summarized in Table 1, highlighting practical teaching points applicable to daily clinical practice.

**Table 1:** Teaching points derived from a case of acute cholecystitis in situs inversus totalis

Teaching Point	References
Left-sided upper abdominal pain does not exclude biliary pathology, particularly in patients with anatomical variants such as situs inversus totalis.	[1,5]
The presence of dextrocardia on chest radiography should prompt further abdominal imaging to evaluate for situs inversus totalis.	[1,5]
Ultrasound and computed tomography are essential for confirming acute cholecystitis and defining anatomical orientation for surgical planning.	[2,6,7]
Situs inversus totalis is not a contraindication to laparoscopic cholecystectomy but requires technical adaptation to mirrored anatomy.	[2,8]
Careful identification of Calot's triangle and strict adherence to surgical safety principles are critical to minimizing bile duct injury.	[2,9,10]

## Conclusions

Acute cholecystitis in the setting of situs inversus totalis represents a diagnostic and surgical challenge due to atypical symptom localization and reversed anatomy. Early recognition of this anatomical variant, combined with appropriate use of imaging, is essential to avoid diagnostic delay and guide safe operative planning. Laparoscopic cholecystectomy can be performed safely with technical adaptation and strict adherence to surgical safety principles. Future research should focus on aggregating multicenter case data to better characterize diagnostic delays, optimize surgical strategies, and establish standardized recommendations for the management of biliary disease in patients with situs inversus totalis.
